# ESR Essentials: staging and restaging with FDG-PET/CT in oncology—practice recommendations by the European Society for Hybrid, Molecular and Translational Imaging

**DOI:** 10.1007/s00330-024-11094-8

**Published:** 2024-10-09

**Authors:** Ricarda Ebner, Gabriel T. Sheikh, Matthias Brendel, Jens Ricke, Clemens C. Cyran

**Affiliations:** 1 Department of Radiology, LMU University Hospital, LMU Munich, Munich, Germany; 2 Department of Nuclear Medicine, LMU University Hospital, LMU Munich, Munich, Germany

**Keywords:** Positron-emission tomography computed tomography, Fluorodeoxyglucose F18, Molecular imaging, Radiopharmaceuticals, Clinical decision-making

## Abstract

**Abstract:**

Positron emission tomography (PET) stands as the paramount clinical molecular imaging modality, especially in oncology. Unlike conventional anatomical-morphological imaging methods such as computed tomography (CT) and magnetic resonance imaging (MRI), PET provides detailed visualizations of internal activity at the molecular and cellular levels.

18-fluorine-fluorodeoxyglucose ([^18^F]FDG)-PET combined with contrast-enhanced CT (ceCT) significantly improves the detection of various cancers. Appropriate patient selection is crucial, and physicians should carefully assess the appropriateness of [^18^F]FDG-PET/CT based on specific clinical criteria and evidence. Due to its high diagnostic accuracy, [^18^F]FDG-PET/CT is indispensable for evaluating the extent of disease, staging, and restaging known malignancies, and assessing the response to therapy. PET/CT imaging offers significant advantages in patient management, particularly by identifying occult metastases that might otherwise go undetected. This can help prevent unnecessary surgeries, allowing many patients to be redirected to systemic chemotherapy instead. However, it is important to note that the gold standard for surgical planning remains CT and/or MRI, depending on the body region. These imaging modalities, with or without associated angiography, provide superior contrast and spatial resolution, essential for detailed surgical preparation and planning.

[^18^F]FDG-PET/CT has a central role in the precise and early diagnosis of cancer, contributing significantly to personalized treatment plans. However, it has limitations, including non-tumor-specific uptake and the potential to inaccurately capture the metabolic activity of certain tumor types due to low uptake in some well-differentiated tumor cell lines. Therefore, it should be utilized in clinical scenarios where it offers crucial diagnostic insights not readily available with other imaging modalities.

**Key Points:**

- *Use [*^*18*^*F]FDG-PET/CT selectively based on clinical appropriateness criteria and existing evidence to optimize resource utilization and minimize patient exposure*.
- *Employ [*^*18*^*F]FDG-PET/CT in treatment planning and monitoring, particularly for assessing chemotherapy or radiotherapy response in FDG-avid lymphoma and solid tumors*.
- *When available, [*^*18*^*F]FDG-PET/CT can be integrated with other diagnostic tools, such as MRI, to enhance overall diagnostic accuracy*.

## Key recommendations

- Proper selection of patients: Before deciding whether or not to perform a [^18^F]FDG-PET/CT, carefully evaluate its appropriateness for the corresponding indication based on specific clinical criteria and existing evidence. [^18^F]FDG-PET/CT should be selectively used in cases where it offers superior diagnostic accuracy compared to conventional imaging in terms of sensitivity, specificity, and impact on clinical management (level of evidence: moderate).
- Use in treatment planning and monitoring: leverage the capabilities of [^18^F]FDG-PET/CT in treatment planning and response monitoring. It has demonstrated significant utility in assessing responses to treatments such as chemotherapy, immunotherapy, or radiotherapy. Additionally, [^18^F]FDG-PET/CT can detect non-responding tumors at an early stage, allowing timely modification of treatment approaches (level of evidence: high).
- Integration with other Diagnostic Tools: When available, [^18^F]FDG-PET/CT can be complemented with other diagnostic modalities to enhance diagnostic accuracy. Additionally, combination with other imaging modalities like MRI complements PET/CT, e.g., providing detailed evaluation of liver metastases (level of evidence: high).

## Introduction

Positron emission tomography (PET) is the most important molecular imaging modality, particularly in the field of oncology. PET differs from conventional anatomical-morphological imaging techniques, such as computed tomography (CT) and magnetic resonance imaging (MRI), in that it provides detailed visualizations of the body’s internal activities at the molecular and cellular levels [1]. However, this advantage comes with a compromise, as molecular imaging usually demonstrates lower spatial resolution compared to CT or MRI.

Fluorodeoxyglucose (FDG) is the most common radiopharmaceutical for cancer imaging. Positron emission tomography with 18-fluorine [^18^F]FDG combined with computed tomography ([^18^F]FDG-PET/CT) has become an established imaging method for detecting various cancers. The half-life of [^18^F], a radioisotope of fluorine that emits positrons, is 110 min, making it feasible to scan patients at sites distant from the cyclotron where [^18^F]FDG is produced.

[^18^F]FDG-PET has a high sensitivity for the detection of tumors but is not tumor-specific. Increased utilization of glucose is characteristic of most cancers, primarily due to the overexpression of membrane glucose transporters (GLUT 1) and elevated expression and activity of glycolytic enzymes, such as hexokinase, compared to non-malignant cells [2].

In this review, we focus on the most prevalent and clinically significant tumors, including lung cancer, lymphoma, head and neck cancer, breast cancer, and colorectal cancer. While [^18^F]FDG-PET/CT can be used to assess a wide range of tumor types, we specifically highlight these cancers due to their high incidence and considerable impact.

### Indications for [^18^F]FDG-PET/CT

Evaluating the extent of disease in staging of the known malignancy is essential for determining the appropriate treatment plan and predicting prognosis. PET imaging is also key to the early detection of (recurrent) tumors in the presence of elevated tumor markers, even when there is no clinical or morphological evidence of disease, allowing for timely intervention. In addition, imaging is helpful in the search for an unknown primary when metastatic disease is the first clinical presentation or when patients present with paraneoplastic symptoms, guiding further diagnostic and therapeutic steps. Conventional imaging techniques provide valuable information to distinguish between benign and malignant lesions, but in approximately one-third of patients, therapeutic management is significantly altered by [^18^F]FDG-PET/CT. Due to significantly higher diagnostic accuracy in the detection of metastatic lesions, PET/CT usually leads to the detection of additional metastasis and consequent upstaging [3].

Furthermore, [^18^F]FDG-PET/CT can play a crucial role in evaluating disease response to chemotherapy, immunotherapy, or radiotherapy. Precise imaging with PET/CT helps to identify optimal sites for biopsy, contributing to an accurate pathological diagnosis. It also assists in planning surgical procedures, allowing for optimized tumor removal [2].

**Fig. 1:**
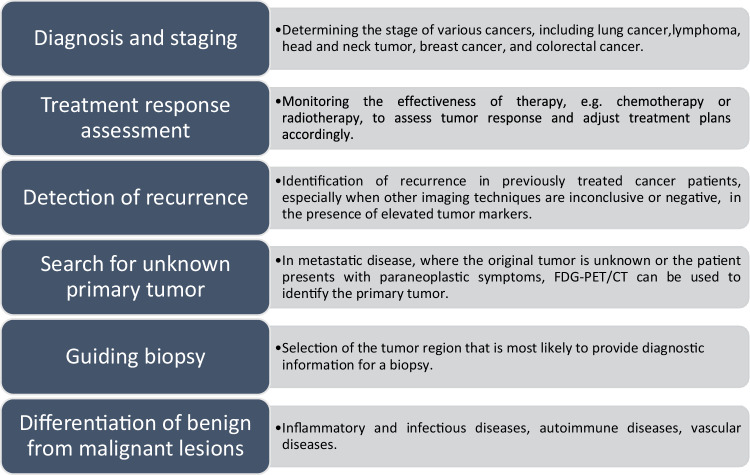
Clinical indications of [^18^F]FDG-PET/CT in oncology

In addition to the acquisition of PET data, a sequential CT scan is usually performed within the same examination. Low-dose CT is primarily used for attenuation correction, while a diagnostic CT scan involves the use of CT with or without intravenous contrast agents (contrast-enhanced CT: ceCT) and helps with the anatomical correlation of PET findings [4]. This diagnostic hybrid imaging approach leverages the combined strengths of complementary morphological and functional imaging techniques. The inclusion of functional information about tumor physiology is essential for a comprehensive assessment of treatment response. While the Response Evaluation Criteria in Solid Tumors (RECIST) criteria primarily focus on anatomical changes, PET Response Evaluation Criteria in Solid Tumors (PERCIST) criteria integrate metabolic activity data from [^18^F]FDG-PET/CT, providing a comprehensive view of tumor response. This combined approach not only enhances the evaluation of therapeutic effectiveness, especially in cases where anatomical changes are minimal but also underscores the importance of structured reporting for referring clinicians, ensuring they receive clear and actionable information for patient management [5]. For most cancer-related scans, covering the area from the base of the skull to mid-thigh is sufficient. However, for patients with a high likelihood of metastases in the lower limbs, a full-body scan might be necessary. Figure 1 illustrates the clinical indications of [^18^F]FDG-PET/CT in oncology.

### Lung cancer

[^18^F]FDG-PET/CT plays a crucial role in lung cancer management, including both small cell lung cancer (SCLC) and non-small cell lung cancer (NSCLC). While solitary pulmonary nodules are often initially detected through plain chest radiographs or CT scans, conventional imaging alone may not definitively determine malignancy due to non-specific anatomical findings. However, [^18^F]FDG-PET/CT is sensitive to increased glucose metabolism associated with cancer before specific structural changes indicate malignancy. For the characterization of solitary pulmonary nodules, [^18^F]FDG-PET/CT demonstrated high sensitivity (96%), accuracy (92%) and low specificity [6]. Due to the low spatial and contrast resolution of PET, primarily resulting from its signal-to-noise ratio and the need for a substantial number of hypermetabolic cells to detect a signal above the background, subcentimeter nodules (< 8–10 mm) can result in false negatives on [^18^F]FDG-PET. However, solid pulmonary nodules larger than 8 to 10 mm without [^18^F]FDG uptake are likely benign [7]. False positives may arise from conditions such as inflammation or infection.

Evidence-based guidelines, for example, the National Comprehensive Cancer Network (NCCN), recommend [^18^F]FDG-PET/CT for initial staging at diagnosis of NSCLC, and for restaging after induction therapy or when recurrence is suspected. This recommendation is based on its higher sensitivity and specificity compared to conventional CT, particularly in staging mediastinal lymph nodes and excluding distant metastases. Accurate staging determines whether patients will undergo surgery or, in cases where surgery is not an option, will benefit from neoadjuvant chemotherapy, radiotherapy alone, or a combination of chemotherapy and radiotherapy.

**Fig. 2:**
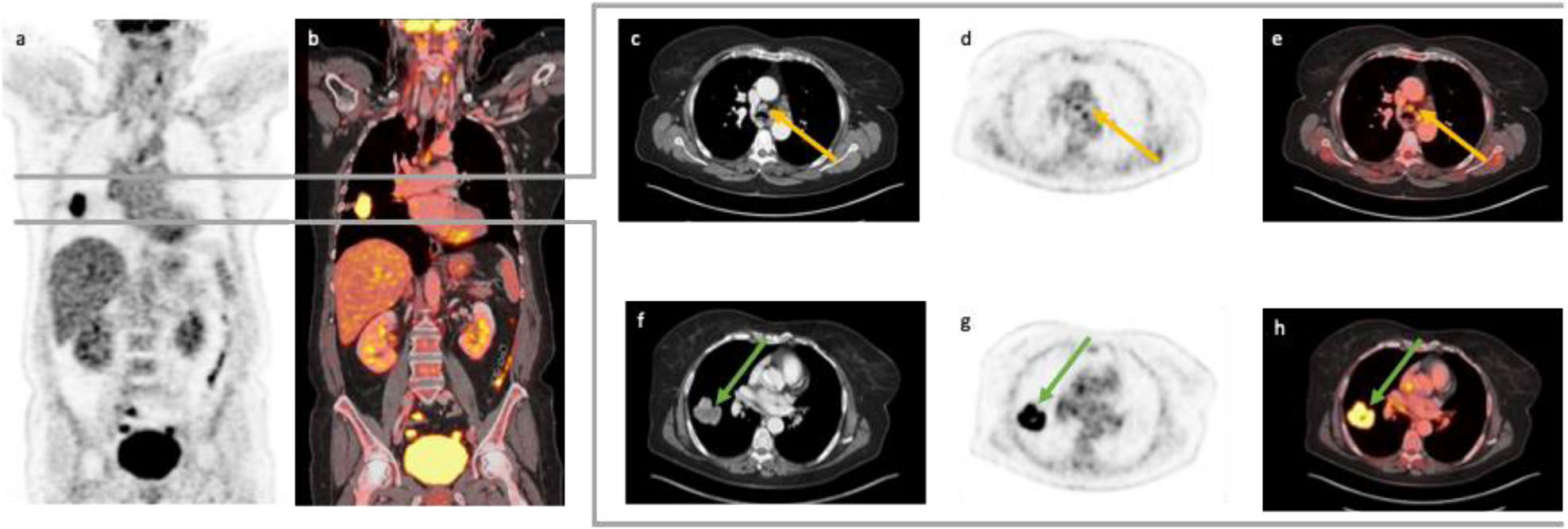
[^18^F]FDG-PET/CT (**a** and **b**) with pulmonary mass in the right upper lobe, suggestive of lung cancer. The pulmonary mass exhibited intense FDG uptake (bottom row **f**–**h**, green arrow). Several mediastinal lymph nodes were identified as pathologic on the CT scan (**c**). However, FDG-PET/ceCT revealed only medium FDG uptake in these lymph nodes (top row **d** and **e**, yellow arrow). A bronchoscopy was recommended for further evaluation, which confirmed no lymph node metastases in the mediastinum

The guidelines also advise its use in accurate radiation therapy planning for both NSCLC and SCLC when limited stage is suspected or when it is necessary to clarify the stage. However, the guidelines do not support the routine use of [^18^F]FDG-PET/CT for follow-up or surveillance in NSCLC or SCLC, despite its superiority in differentiating benign conditions such as atelectasis, consolidation, and radiation fibrosis from neoplasms, compared to conventional CT. Although [^18^F]FDG-PET/CT can be effective in these cases, it requires histopathologic confirmation of recurrence, as post-radiation changes can remain FDG-avid for up to 2 years [8, 9]. For detailed visualization, Fig. 2 displays [^18^F]FDG-PET/ceCT imaging of a pulmonary mass in the right upper lobe, indicative of lung cancer.

### Lymphoma

In the diagnosis and treatment of hematologic malignancies, [^18^F]FDG-PET/CT is essential for initial staging, assessing therapeutic responses, and detecting potential recurrence. [^18^F]FDG-PET/CT is recommended in evidence-based guidelines for the initial staging of FDG-avid lymphomas, such as Hodgkin’s lymphoma, diffuse large B-cell lymphoma (DLBCL), and follicular lymphoma. [^18^F]FDG-PET/CT is more accurate than CT in staging aggressive lymphomas with significantly higher sensitivity and specificity, especially in assessing lymphoma viability and in the detection of extranodal disease. Guidelines state that [^18^F]FDG-PET/CT is useful for detecting histologic transformation from follicular lymphoma to DLBCL, as transformed lymphomas often show higher FDG uptake values [10].

The initial staging and treatment response of lymphoma can be objectively assessed using the Deauville five-point scale. The scoring system assigns a numerical value to FDG uptake in lymph nodes or other sites of disease. It provides a standardized approach for interpreting [^18^F]FDG-PET/CT images, allowing for consistent assessment of treatment response across different centers and over time. However, it is essential to consider clinical context and other imaging findings besides the Deauville score to ensure accurate interpretation and appropriate clinical decision-making [11].

Following the early evaluation of chemotherapy response, typically after two or three cycles, decisions regarding treatment escalation or de-escalation can be made based on the results of [^18^F]FDG-PET/CT [12]. Additionally, PET scans are valuable at the end of chemotherapy for assessing residual masses on CT by determining their metabolic activity and guiding decisions on the necessity of further treatment.

The German Hodgkin Study Group Hodgkin disease (HD)15 and HD18 studies significantly impacted lymphoma treatment protocols by using PET scans to guide therapy adjustments. The HD15 study reduced the chemotherapy regimen in patients with advanced-stage Hodgkin’s lymphoma from eight to six cycles for better efficacy and reduced toxicity. It also determined that patients with PET-negative residual lymphomas post-chemotherapy had a prognosis as favorable as those with a complete response. Only patients with PET-positive residuals were advised to undergo localized 30 Gray radiation [13].

The HD18 study tested further reduction of chemotherapy cycles for patients in patients with advanced-stage Hodgkin’s lymphoma responding well early on. Results showed that patients who were PET-negative after two cycles of escalated BEACOPP could safely reduce their treatment to four cycles without losing tumor control, with overall survival improving significantly. Hence, for advanced stages, patients with a negative PET after two cycles now receive only four cycles of escalated BEACOPP, while those with PET-positive residual manifestations continue with six cycles and may require radiation therapy [14].

Chimeric antigen receptor (CAR T)-cell therapy represents an innovative advancement in the treatment of hematologic malignancies, using the patient’s own immune system to effectively target cancer cells. Acute lymphocytic leukemia, non-Hodgkin lymphoma (NHL) and multiple myeloma are particularly difficult to manage, especially when they relapse after initial treatments. CAR T-cell therapy offers a substantial overall response rate of up to 80%, achieving long-lasting remissions or potential cures in 40 to 50% of cases.

**Fig. 3:**
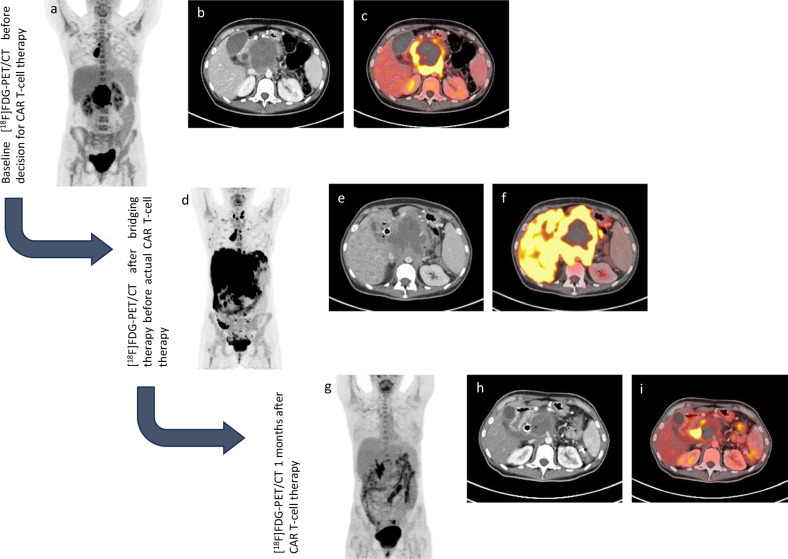
Following initial chemotherapy and first response, the patient with NHL demonstrated disease progression on subsequent follow-up evaluation (**a**–**c**). Consequently, an indication for chimeric antigen receptor (CAR) T-cell therapy was established. Prior to the infusion of CAR T cells, the patient was commenced on R-CHOEP as bridging therapy. Despite this regimen, there was a further progression of the disease, evidenced by increasing nodal involvement and new extranodal manifestations (liver, **d**–**f**). One month post-CAR T-cell therapy, imaging revealed only minimal residual nodal disease, and both functional and morphological regression of the hepatic lesions (**g**–**i**)

[^18^F]FDG-PET/CT imaging plays a crucial role in the evaluation and management of patients undergoing CAR T-cell therapy. Two [^18^F]FDG-PET/CT scans should be performed before CAR T-cell infusion: one at the time of the decision to proceed with CAR T-cell therapy, providing a new baseline study, identifying patients who would benefit most, and deciding on aggressive bridging treatments. Another PET/CT study should be conducted after the completion of bridging therapy, which may include treatments such as steroids, systemic therapy, or radiotherapy, administered between T-cell harvesting and CAR T-cell infusion. High metabolic activity in the disease at baseline is linked to shorter overall survival, higher tumor burdens associated with early relapse and lower burdens with longer survival and progression-free periods. Post-therapy [^18^F]FDG-PET/CT scans at 1 and 3 months post-infusion assess treatment response, nonresponse and treatment failure, classified as early or late depending on whether it occurs within or after 90 days post-treatment [15, 16]. Figure 3 illustrates the disease progression and therapeutic responses in a patient with NHL following initial chemotherapy, highlighting the effectiveness of subsequent CAR T-cell therapy.

### Head and neck tumors

[^18^F]FDG-PET/CT is recommended for various clinical applications in head and neck cancer, including staging, identifying lymph node and distant metastases, and detecting unknown primary tumors. It offers high sensitivity and specificity, both over 90% [17]. Furthermore, hybrid imaging aids in detecting recurrent head and neck tumors, especially when postradiogenic or postoperative changes complicate interpretation in morphological CT and MRI. [^18^F]FDG-PET/CT is superior to CT and MRI in differentiating tumor recurrence from postradiogenic or postoperative inflammation or defects. Other guidelines recommend restaging with [^18^F]FDG-PET/CT 3 months after surgery, with additional imaging only if symptoms arise or if results from conventional imaging are inconclusive. In advanced head and neck tumors that cannot be treated surgically, [^18^F]FDG-PET/CT should be considered 3–6 months after systemic treatment to assess residual disease. Earlier scans, which might yield false-positive results, should be avoided [18].

### Breast cancer

[^18^F]FDG-PET/CT is not routinely used for the early diagnosis of breast cancer, since it has low sensitivity for local lesions below 5 mm (< 50%) and demonstrates only moderate diagnostic accuracy in axillary staging, but it can be very useful in detecting distant metastases [19].

**Fig. 4:**
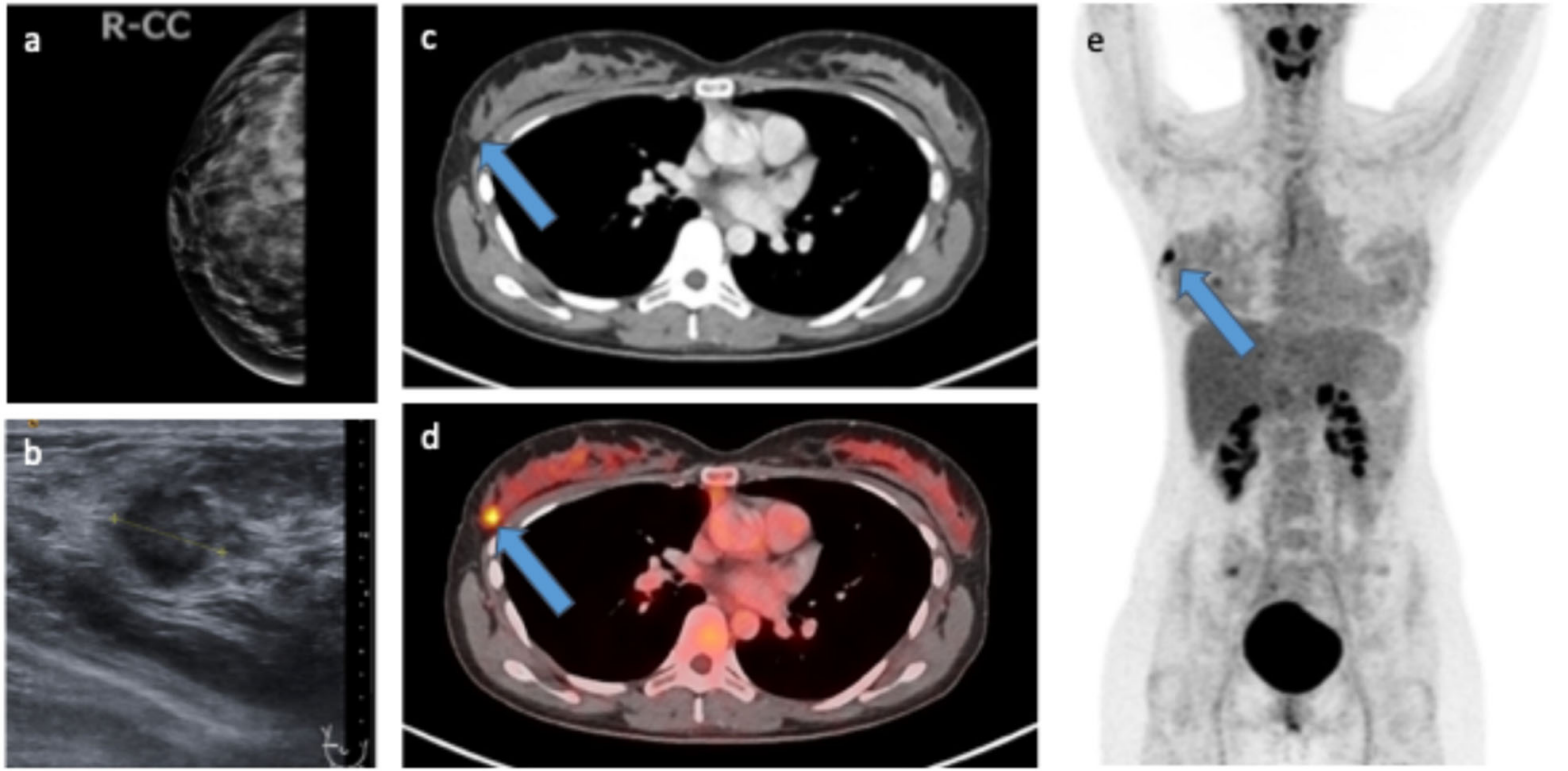
Due to the dense breast tissue (**a**), no suspicious lesions were detected on mammography. Ultrasonography of the right breast measured a 1.5 cm lesion (**b**). Given the patient’s young age (aged 30) and clinical suspicion of metastases, FDG-PET/CT was performed (**c**–**e**). FDG-avid lesion in the right lower outer quadrant (**d**) was not clearly identified on CT (**c**). The right axillary lymph nodes were not enlarged and showed no suspicious FDG uptake. The maximum intensity projection also shows a singular lesion in the right breast (**e**). No additional lesions suspicious for metastases were detected

The initial staging process in suspected breast cancer should involve mammography, the reference standard for detecting primary breast tumors, and, where indicated, MRI of the breast, as well as ultrasonography of the breast and axilla [20]. According to the NCCN guidelines, staging with body CT, bone scintigraphy, and optional [^18^F]FDG-PET/CT is recommended for signs or symptoms of possible metastases, stage IV disease, inflammatory breast cancer, more than four positive axillary nodes at surgery, and workup before preoperative systemic therapy [21]. Routine systemic staging is not recommended for early breast cancer in the absence of symptoms. [^18^F]FDG-PET/CT can be considered for staging of recently diagnosed stage III and, in some cases, stage IIB breast cancer. Due to its limited sensitivity in detecting early axillary lymph node disease and micro metastases, [^18^F]FDG-PET/CT cannot replace sentinel lymph node biopsy (SLNB) for staging. Precise staging, particularly in the axillary lymph nodes, is crucial for assessing patient prognosis and selecting appropriate (multimodality) treatment strategy. SLNB remains the reference standard for lymph node staging. However, in 25% of breast cancer patients who underwent [^18^F]FDG-PET/CT, significant changes in staging were observed, and 18% experienced changes in treatment [22]. Figure 4 illustrates the diagnostic imaging sequence for a young patient with dense breast tissue, where mammography did not reveal any suspicious lesions, but subsequent ultrasonography and FDG-PET/CT identified and characterized a single lesion in the right breast.

The common sites of distant metastasis in breast cancer are bone, lung, liver, and brain with a sensitivity of 96% and a specificity of 95% for the detection of distant metastasis by [^18^F]FDG-PET/CT [23]. [^18^F]FDG-PET/CT has demonstrated superior diagnostic accuracy in the detection of frequently CT-occult bone metastasis, achieving a sensitivity of 93% and a specificity of 99%, compared to bone scintigraphy [24].

[^18^F]FDG-PET/CT is not a standard procedure for the routine follow-up of patients with breast cancer but is highly recommended in asymptomatic patients with rising tumor markers (cancer antigen [CA 15-3], carcinoembryonic antigen, or CA 125), especially if the results of conventional images are inconclusive [25].

### Colorectal cancer

Over 75% of colorectal cancer (CRC) patients present with the disease limited to the bowel or regional pericolic or mesenteric lymph nodes at the initial diagnosis. Typically, treatment in the early stages involves surgical removal with curative intent. A subset of patients (Union for International Cancer Control: UICC stage III) receive adjuvant chemotherapy. The spread to locoregional lymph nodes serves as an important prognostic factor. Five-year survival rates of 80% in UICC stage II and 45–50% in UICC stage III. Accurate preoperative staging is crucial for assessing prognosis and establishing an appropriate, potentially curative therapy regimen [26].

Imaging techniques such as endoscopic ultrasound and MRI are preferred for locoregional staging. The current standard for staging and monitoring recurrence in CRC patients generally relies on conventional imaging techniques, such as contrast-enhanced CT of the chest, abdomen, and pelvis. However, CT generally has a high false-negative rate for extrahepatic intra-abdominal lesions, such as paraaortic lymph nodes, and a high false-positive rate for pulmonary lesions. [^18^F]FDG-PET/CT provides additional functional information on tumor glucose metabolism, leading to higher diagnostic accuracy for initial staging, detection of nodal and extranodal metastases, therapy monitoring, and assessment of recurrence [27].

According to evidence-based guidelines for CRC, CT is considered the imaging first-choice procedure. However, the guidelines also recommend that in cases where liver-directed treatment or surgery is being considered, MRI of the liver with intravenous contrast, using either standard or hepatobiliary agents, is preferred over CT and [^18^F]FDG-PET/CT, to assess the exact number and distribution of metastases for local treatment planning [28]. MRI is considered a cost-effective strategy for detecting liver metastases suitable for hepatic resection, making it the preferred imaging modality in diagnostic workup [29].

The role of [^18^F]FDG-PET/CT in the staging of CRC is limited by several factors. The ability to detect small or early-stage tumors is impaired by the limited resolution of PET, as lesions smaller than 5 mm cannot usually be clearly detected. The supplementary information provided by [^18^F]FDG-PET/CT may not influence treatment decisions in early colorectal cancer, so its application is generally reserved for certain conditions where its detailed diagnostic insight offers clear advantages, such as assessing the spread of metastases or response to treatment [27].

## Conclusion

[^18^F]FDG-PET/CT demonstrates a well-documented higher diagnostic accuracy for staging of most malignant tumors, however indications for [^18^F]FDG-PET/CT need to be carefully selected depending on the clinical risk profile to fully maximize its added value.

Despite its strengths, [^18^F]FDG-PET/CT is not without limitations. Its spatial resolution is about several millimeters, and it may not accurately capture the metabolic activity of certain tumor types. Furthermore, benign conditions involving inflammation or infections can also exhibit increased FDG uptake, necessitating cautious interpretation of results.

The future of PET/CT involves technological advances aimed at reducing the radiation dose and improving resolution. The PET/MR system offers high soft tissue contrast with no additional ionizing radiation from the MR component, making it particularly suitable for pediatric and nononcologic patients. However, PET/MR has longer examination times, lower throughput, and its clinical use is currently limited, though ongoing research aims to expand its applications, including combinations with functional MRI techniques [30].

Current innovations in cancer imaging with PET/CT are focusing on the transition from the widely used [^18^F]FDG tracer to innovative, cancer- and receptor-specific tracers. [^68^Ga] and [^18^F]–labeled fibroblast activation protein inhibitor (FAPI) PET serves as an advanced imaging technique that targets cancer-associated fibroblasts within tumor stroma, which are known for their high expression of FAP. Furthermore, FAPI can be utilized in rheumatological disorders for enhanced precision and disease management. FAPI stands out due to its rapid accumulation in tumors and minimal background interference, leading to superior imaging quality in some malignancies. This radiotracer is particularly effective in detecting small primary or metastatic lesions in areas like the brain, liver, pancreas, and gastrointestinal tract that show high physiological [^18^F]FDG accumulation. Thus, FAPI PET imaging may also have the potential to depict several common benign disease processes that are associated with widespread morbidity [31].

Beyond that, prostate-specific membrane antigen PET/CT is particularly effective in the detection and staging of prostate cancer, while somatostatin receptor-PET/CT is highly effective for imaging neuroendocrine tumors, offering high sensitivity and specificity. Its clinical utility has been well-documented, making it a standard for prostate cancer and neuroendocrine tumor imaging [32, 33]. [^18^F]-fluoroestradiol (FES) is another notable radiotracer, primarily used in breast cancer imaging. FES is utilized in clinical settings for patients with estrogen receptor (ER)-positive recurrent or metastatic breast cancer and as an adjunct to biopsy. It binds to ER, allowing for a comprehensive in vivo assessment of ER expression throughout the body [34].

These recent developments represent a significant advancement in molecular medicine and imaging, offering enhanced disease detection compared to conventional methods. This innovation is transforming patient care worldwide by improving the accuracy of staging and disease management in about one-third of cases. These innovations promise to further refine diagnostic and therapeutic strategies, keeping hybrid imaging at the forefront of cancer treatment.

### Summary statement

PET with [^18^F]FDG, particularly when integrated with ceCT is a highly valuable molecular imaging technique in oncology. This technology combines the functional insight of PET imaging, which highlights the metabolic activity of cells, with the anatomical detail provided by CT, offering therapeutic guidance for cancer diagnosis, staging, and monitoring of treatment response. Despite its extensive utility, PET faces limitations such as comparably low spatial resolution in relation to CT or MRI, and the non-specific uptake of [^18^F]FDG that may lead to false-positive results in inflammatory or infectious conditions. Moreover, while PET/CT is instrumental in certain clinical scenarios, its high cost and limited availability restrict its use as a routine first-line imaging tool, especially for early-stage cancer. Therefore, PET/CT is often reserved for specific indications where its detailed metabolic information can significantly influence clinical decisions.

### Patient summary

PET/CT imaging is an advanced diagnostic tool that combines the detailed anatomical depiction of CT with the metabolic and functional information provided by PET. This establishes PET/CT as an invaluable resource in the detection and management of cancer.

Utilized not only for identifying the presence of cancer, PET/CT scans are helpful in monitoring the effectiveness of ongoing treatments and in detecting the recurrence of disease after therapy. This innovative technology plays a crucial role in devising personalized treatment plans, ensuring that each patient receives the most effective and targeted therapy available, thereby enhancing the chance of successful outcomes and improved quality of life.

## Abbreviations

BEACOPP: Cancer drug combination of Bleomycin, Etoposide, Adriamycin, Cyclophosphamide, Vincristine, Procarbazine, Prednisolone
CA: Cancer antigen
CAR T: Chimeric antigen receptor
ceCT: Contrast-enhanced computed tomography
CRC: Colorectal cancer
CT: Computed tomography
DLBCL: Diffuse large B-cell lymphoma
F: Fluorine
FAPI: Fibroblast activation protein inhibitor
FDG: Fluorodeoxyglucose
FES: Fluoroestradiol
HD: Hodgkin disease
MRI: Magnetic resonance imaging
NCCN: National Comprehensive Cancer Network
NHL: Non-Hodgkin lymphoma
NSCLC: Non-small cell lung cancer
PET: Positron emission tomography
R-CHOEP: Cancer drug combination of Rituximab, Cyclophosphamide, Hydroxydaunorubicin, Vincristine, Prednisolone
RECIST: Response Evaluation Criteria In Solid Tumors
SCLC: Small cell lung cancer
SLNB: Sentinel lymph node biopsy
UICC: Union for International Cancer Control
